# Multi-Scale Graph Representation Learning for Autism Identification With Functional MRI

**DOI:** 10.3389/fninf.2021.802305

**Published:** 2022-01-13

**Authors:** Ying Chu, Guangyu Wang, Liang Cao, Lishan Qiao, Mingxia Liu

**Affiliations:** ^1^School of Mathematics Science, Liaocheng University, Liaocheng, China; ^2^Department of Information Science and Technology, Taishan University, Taian, China; ^3^Taian Tumor Prevention and Treatment Hospital, Taian, China

**Keywords:** functional connectivity, graph convolutional networks, autism, resting-state functional MRI, classification

## Abstract

Resting-state functional MRI (rs-fMRI) has been widely used for the early diagnosis of autism spectrum disorder (ASD). With rs-fMRI, the functional connectivity networks (FCNs) are usually constructed for representing each subject, with each element representing the pairwise relationship between brain region-of-interests (ROIs). Previous studies often first extract handcrafted network features (such as node degree and clustering coefficient) from FCNs and then construct a prediction model for ASD diagnosis, which largely requires expert knowledge. Graph convolutional networks (GCNs) have recently been employed to jointly perform FCNs feature extraction and ASD identification in a data-driven manner. However, existing studies tend to focus on the single-scale topology of FCNs by using one single atlas for ROI partition, thus ignoring potential complementary topology information of FCNs at different spatial scales. In this paper, we develop a multi-scale graph representation learning (MGRL) framework for rs-fMRI based ASD diagnosis. The MGRL consists of three major components: (1) multi-scale FCNs construction using multiple brain atlases for ROI partition, (2) FCNs representation learning via multi-scale GCNs, and (3) multi-scale feature fusion and classification for ASD diagnosis. The proposed MGRL is evaluated on 184 subjects from the public Autism Brain Imaging Data Exchange (ABIDE) database with rs-fMRI scans. Experimental results suggest the efficacy of our MGRL in FCN feature extraction and ASD identification, compared with several state-of-the-art methods.

## 1. Introduction

Autism spectrum disorder (ASD) is a developmental disorder that can cause major social, communication, and behavioral challenges (Simonoff et al., 2008). In 2014, the overall prevalence of autism was estimated at 16.8 per 1, 000 8-year-old children, and the prevalence of ASD reached nearly 3% in some communities (Baio et al., 2018). In recent years, people have been worried about the increased prevalence of ASD in children (Hodges et al., 2020; Ahammed et al., 2021). However, the current diagnosis of autism is highly dependent on traditional behavioral symptoms, which are usually subjective and can easily lead to neglect early symptoms and misdiagnosis (American Psychiatric Association, 2013; Lord et al., 2018). Therefore, seeking an objective biomarker for early diagnosis and timely intervention in the treatment of autism has attracted increasing attention in the field of psychiatry and neuroscience.

Resting-state functional MRI (rs-fMRI) is a technique to measure a subject's blood-oxygen-level-dependent (BOLD) signals without performing any specific task and has been widely used in neuroimaging analysis (Buckner et al., 2013; Li et al., 2020). With rs-fMRI, the functional connectivity networks (FCNs) are usually constructed for representing each subject, with each element representing the pairwise relationship between brain region-of-interests (ROIs) (Dvornek et al., 2017; Xing et al., 2019). Therefore, FCNs tend to capture the dependencies between BOLD signals of paired region-of-interests (ROIs) of the brain and have been used to identify potential neuroimaging biomarkers for the diagnosis of neurological diseases (El Gazzar et al., 2019; Wang et al., 2019a). It can help us understand brain organization patterns and diagnose neurological diseases such as ASD (Bijsterbosch and Beckmann, 2017; Kazi et al., 2019), Alzheimer's disease and its prodromal stage (i.e., mild cognitive impairment) (Amini et al., 2021), Parkinson's disease (Vivar-Estudillo et al., 2021). However, previous studies often first extract handcrafted network features (such as node degree and clustering coefficient) from FCNs and then conduct prediction models for ASD diagnosis (Wang et al., 2019b), where these two steps are treated separately and highly rely on expert knowledge.

With the development of deep learning, especially graph neural networks (GNNs) have been developed to identify potential fMRI biomarkers in brain FCNs for disease diagnosis (Li et al., 2019; Wu et al., 2020). In general, each brain network can be viewed as a complex graph structure composed of irregular data (Zhang et al., 2018a), containing not only node features but also topology information among different nodes. Graph convolutional networks (GCNs) provide an end-to-end deep learning framework to automatically learn node features and topology information between nodes. Current studies have shown that the application of GCNs in fMRI analysis helps automatically capture the high-level topological information of brain networks through operations, such as convolution and graph pooling, thus significantly improving the diagnosis performance of brain diseases (Yu et al., 2020). To facilitate functional connectivity (FC) analysis and computational modeling of human brain functions, existing studies generally partition each brain into multiple ROIs (Dvornek et al., 2017; Xing et al., 2019), followed by GCN models for FCNs feature learning and disease diagnosis. However, previous studies usually focus on single-scale topology of FCNs by using one single atlas for brain ROIs partition (Chen et al., 2017; Wang et al., 2018), thus ignoring the potential complementary topology information of FCNs at different spatial scales.

**Figure 1:**
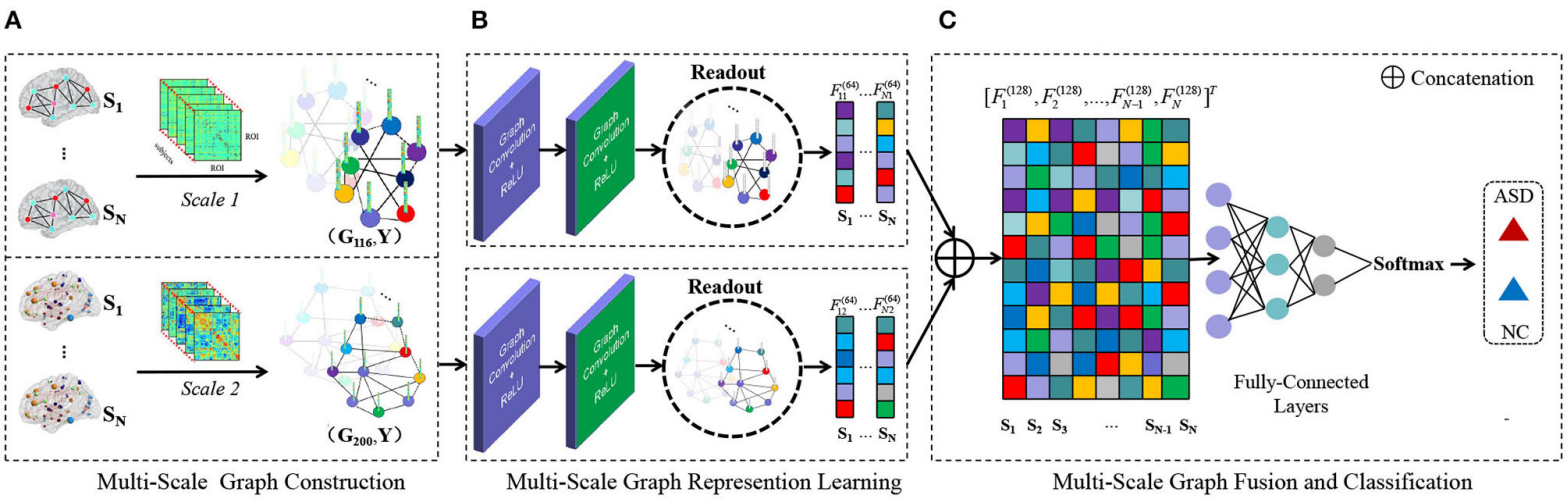
Illustration of the proposed multi-scale graph representation learning (MGRL) framework for autism spectrum disorder (ASD) identification, consisting of three components. **(A)** Multi-scale graph construction. Let's say there are *N* subjects in the classification task. First, think of the brain (region of interests, ROIs) as nodes on a graph. Based on the Automated Anatomical Labeling (AAL) atlas and Craddock200 (CC200) atlas, 2*N* functional connectivity networks (FCNs) are constructed by Pearson's correlation (PC). PC coefficient matrix can represent the paired similarity relation between brain regions, so as to construct weighted edge connection of brain graphs and establish a multi-scale graph of each subject. (*G*_116_, *Y*) and (*G*_200_, *Y*). Subscripts 116, 200 represent different brain atlases, and *Y*∈{0, 1} represents the label of the subject (with 0 indicating NC and 1 representing ASD). **(B)** MRGL. The graph structure and node content information of different scales from the same subject are used as input of Graph convolutional networks (GCN) to train independent GCN models. Convolution operation and readout operation are used to automatically learn graph representation vectors of different scales. **(C)** Multi-scale feature fusion and classification. Read higher-order graph representation vectors are spliced together to integrate complementary information at the multi-scale graph. The final classification results are obtained through three fully-connected layers and a softmax layer for classification.

To this end, we develop a multi-scale graph representation learning (MGRL) framework for rs-fMRI based ASD diagnosis. As shown in Figure 1, we first construct multi-scale graphs (with each graph corresponding to a specific FCN) for each subject, by partitioning the brain into multiple ROIs using two atlases, i.e., Automated Anatomical Labeling (AAL) (Tzourio-Mazoyer et al., 2002) atlas with 116 ROIs and Craddock200 (CC200) (Craddock et al., 2012) atlas with 200 ROIs. Then, we propose to learn multi-scale graph representations via GCNs for each subject, followed by multi-scale features fusion. The fused features are finally fed into three fully-connected layers and a classification layer (via Softmax) for disease diagnosis. The proposed MGRL allows the automated integration of fine-grained topology information of FCNs at different spatial scales. Experiments on 184 subjects with rs-fMRI data from the Autism Brain Imaging Data Exchange (ABIDE) database suggest that the MGRL helps improve the performance of ASD diagnosis, compared with several state-of-the-art methods.

The rest of this paper is organized as follows. In section Related Work, we review the most relevant studies. In section Materials and Methods, we describe the data set used in this study and the proposed method. We then present experimental settings, competing methods, and results of ASD diagnosis achieved by different methods in section Experiments. In section Discussion, we investigate the influence of several major components of the proposed MGRL method and discuss the limitations of the current work and several possible future research directions. Finally, this paper is concluded in section Conclusion.

## 2. Related Work

In this section, we first briefly introduce the most relevant studies on features extraction of FCNs based on rs-fMRI and then introduce existing GCN based methods for computer-aided disease diagnosis.

### 2.1. Feature Extraction of Functional Connectivity Networks (FCNs)

Functional MRI has been widely used to establish brain FCNs (Yu et al., 2019; Xue et al., 2020) by focusing on measuring the FC between two network nodes. Identifying distinguishable and explainable features from FCNs is essential for subsequent classification/regression tasks and helps us understand the pathological mechanisms of related brain disorders. Previous studies usually extract node statistics or edge weights from functional brain networks to represent each subject and mine the correlation of temporal and spatial information between brain regions. For example, Chen et al. used Pearson's correlation (PC) coefficient to compute edge weights for FCNs construction (Chen et al., 2020). Hhimilon et al. extracted both global and node-level statistics (such as local clustering coefficients) as FCN attributes (Hamilton, 2020). Jie et al. extracted local clustering coefficients from hyperconnected FCNs to represent each FCN for disease diagnosis (Jie et al., 2016). Recently, Zhang et al. proposed a modularity-based feature selection method to identify discriminative and interpretable features from functional brain networks for the diagnosis of Alzheimer's disease (AD) and related disorders (Zhang et al., 2021).

Although previous studies have yielded promising results, topological measures involved in these methods require manual design (i.e., manual definition of FCNs features), which is usually subjective and largely relies on expert knowledge. Besides, Mangor et al. found that handcrafted features (e.g.„ median centrality) of individuals with focal epilepsy did not differ significantly between patients and healthy controls and could not be discriminative for disease classification (Pedersen et al., 2015). This implies that the handcrafted FCNs features could be suboptimal for diagnosis, due to the fact that these features are extracted independently from subsequent classification models.

### 2.2. GCN for fMRI Analysis

With the development of deep learning techniques, GCNs have been increasingly employed to model topological information of brain FCNs (Anirudh and Thiagarajan, 2019). Significant progress has been made in early intervention of neurological diseases. Song et al. extracted the mean rs-fMRI time series of a set of 90 ROIs based on the AAL atlas, and then proposed similarity-aware adaptive calibrated GCN to predict significant memory concern and MCI (Song et al., 2021). Wang et al. constructed FCNs based on the Power atlas (Power et al., 2011) and utilized a GCN model to extract the spatial characteristics of linogroups from rs-fMRI data for ASD classification (Wang et al., 2021). Ktena et al. extracted brain time series based on Harvard Oxford (HO) (Craddock et al., 2012) atlas and proposed to use Siamese GCN (SGCN) to analyze the brain FCNs of autism classification (Ktena et al., 2018).

These deep learning methods show excellent performance in automated FCNs features extraction, and some of them have realized that fine-grained and coarse-grained topological properties of FCNs at different spatial scales may affect the final performance. However, existing GCN-based studies tend to extract single-scale representations of FCNs by using one single atlas for brain ROIs partition. This will ignore the potential complementary topological information conveyed by multi-scale brain atlases, thereby reducing the learning performance of the prediction/diagnostic model. Intuitively, it is interesting to model multi-scale representations of brain FCNs to improve the performance of ASD diagnosis. In this work, we will develop an MGRL framework to capture multi-scale topological features of FCNs for automated ASD identification.

## 3. Materials and Methods

In this section, we will first introduce the data set and image preprocessing steps used in this study. Then, we will introduce the proposed MGRL framework and the implementation details.

### 3.1. Subjects and Image Processing

The ABIDE (Di Martino et al., 2014) includes baseline resting-state fMRI data from patients with ASD and normal controls (NC). In this work, we use rs-fMRI data from the New York University (NYU) site with the largest sample size collected in this database. Specifically, the NYU site includes 184 subjects, 79 of whom were from ASD and 105 NC cases. We report the phenotype information of the studied subjects in Table 1. All fMRI data involved are provided by the Preprocessed Connectome Project initiative. The 3.0 Tesla Allegra scanner is used to collect data, and the imaging parameters are set as follows: the number of slices is 33, and TR/TE is 2, 000/15 ms with 180 volumes. Then, the remaining volumes are processed by a well-accepted pipeline with the Data Processing Assistant for Resting-State fMRI toolbox (DPARSF) (Yan et al., 2016). Specifically, the preprocessed pipeline primarily includes the following: (1) head motion correction, (2) nuisance signals regression (ventricle, cerebrospinal fluid (CSF), white matter signals and the high-order effect of head motion described by Friston 24-parameters model), (3) spatial standardization of the Montreal Neurological Institute (MNI) template (Tzourio-Mazoyer et al., 2002), 3 × 3 × 3*mm*^3^ resolution, and (4) time-high pass filtering (0.01–0.10*Hz*) based on a linear downtrend and fast Fourier transform. Then, each brain is partitioned into 116 and 200 ROIs based on multi-scale atlases, i.e., AAL atlas and CC200 atlas, respectively. Finally, the extracted mean time series from all these ROIs are put into the data matrix *S*∈*R*^(175 × *n*)^ (*n* = 116 or *n* = 200).

**Table 1:** Demographic information of the subjects in New York University (NYU) site and the University of Michigan (UM) site of the Autism Brain Imaging Data Exchange (ABIDE) dataset.

**Dataset**	**Category**	**Gender (M/F)**	**Age**	**FIQ**
NYU	ASD (*N* = 79)	68/11	14.51 ± 6.23	107.92 ± 3.15
	NC (*N* = 105)	79/26	15.80 ± 3.23	113.15 ± 2.45
UM	ASD (*N* = 68)	58/10	13.13 ± 2.41	105.46 ± 17.28
	NC (*N* = 77)	59/18	14.79 ± 3.57	108.12 ± 9.80

*Values are reported as mean ± SD. M/F, Male/Female; FIQ, Full-scale intelligence quotient; ASD, Autism spectrum disorder; NC, Normal control; ABIDE, Autism brain imaging data exchange*.

### 3.2. Proposed Method

We attempt to solve two challenging problems in fMRI-based FCNs analysis: (1) how to model multi-scale topological information of brain FCNs, and (2) how to integrate these multi-scale FCNs features for ASD diagnosis. To this end, we develop a multi-scale graph representation learning (MGRL) framework to first extract brain FCNs features at multiple scales and then fuse them for automated brain disease identification. The MGRL framework consists of three main components: (1) multi-scale graph construction; (2) multi-scale graph representation learning; and (3) multi-scale feature fusion and classification.

#### 3.2.1. Multi-Scale Graph Construction

Let *G* = {*V, E, A*} denotes an undirected graph with *n* nodes/ROIs, where *V* (||*V*|| = *n*) is a set of nodes and *E*∈ℝ^*n*×*n*^ is a set of edges, and *A*∈ℝ^*n*×*n*^ represents an adjacency matrix corresponding to a specific FCN. The adjacency matrix defines the interconnections between nodes/ROIs.

Craddock et al. found that when the brain is divided into about 200 brain regions, the ROI obtained is appropriately large, which can adapt to individual anatomical variation (Craddock et al., 2012). Wang et al. also proposed that aMCI and NC can be better distinguished when the number of segmented brain regions is appropriate (Wang et al., 2013). In this study, we use AAL atlas and CC200 atlas, which are widely used to locate brain active regions in functional neuroimaging studies, to obtain ROIs time series at different spatial scales. Thus, for each subject, we have two FC matrices established on two different scales. For each FC matrix, each connectivity represents the PC of the mean time series signals between a pair of ROIs. The edge weight *e*_*ij*_∈[−1, 1] between the *i*-th and *j*-th ROIs is defined as follows:


(1) $$\begin{array}{l} e_{ij}=\frac{{\left(s_{i}-{\bar{s}}_{i}\right)}^{T}\left(s_{j}-{\bar{s}}_{j}\right)}{\sqrt{{\left(s_{i}-{\bar{s}}_{i}\right)}^{T}\left(s_{i}-{\bar{s}}_{i}\right)}\sqrt{{\left(s_{j}-{\bar{s}}_{j}\right)}^{T}\left(s_{j}-{\bar{s}}_{j}\right)}} \end{array}$$

Where, $s_{i}\in {\mathbb{R}}^{t}$ represents the time series of BOLD signals extracted from the *i*-th ROI. *t* represents the number of time points in the average time series of each ROI, ${\bar{s}}_{i}$ is the mean vector corresponding to *s*_*i*_.

We describe the features of each node/region in the brain network through the correlation coefficient (i.e., the edge weight *e*_*ij*_). All subjects are divided into 116 ROIs by AAL atlas and 200 ROIs by CC200 atlas. Therefore, the similarity between the *i*-th brain region and other brain regions constitutes the *n*-dimensional feature vector (*e*_*i*1_, *e*_*i*2_, ⋯ , *e*_*in*_), *n* = 116 or *n* = 200. Then, for the same subject, we will get two feature matrices *X*^(116)^∈*R*^116 × 116^, *X*^(200)^∈*R*^200 × 200^. At the same time, we define *X*∈{*X*^(116)^, *X*^(200)^}.

We use the connection strength between the current ROI and other ROIs to measure the edge of the FCNs. The assumption that the edge weight is non-negative conforms to the structural equilibrium theory (Heider, 1946; Cartwright and Harary, 1956). That is, if and only if the edge weights of all edges are positive, the estimated network structure is balanced. In addition, this assumption can simplify subsequent FCNs analysis and convolution operations, and many FC indicators, such as mutual information (Salvador et al., 2007), are also non-negative. Here, we assume that the strength of marginal connections between brain regions, whether the positive correlation of promotion or negative correlation of inhibition, is measured by the value of marginal weight (*e*_*ij*_). Then, the connection strength between the *i*-th brain region and other brain regions will form the *n*-dimensional vector (|*e*_*i*1_|, |*e*_*i*2_|, ⋯ , |*e*_*in*_|), *n* = 116 or *n* = 200. Therefore, for the same subject, the adjacency matrices *A*^(116)^∈*R*^116 × 116^ and *A*^(200)^∈*R*^200 × 200^ for two spatial scales will be defined. At the same time, we define *A*∈{*A*^(116)^, *A*^(200)^}. Finally, two graphs [i.e., $G_{116}=G\left(X^{\left(116\right)},A^{\left(116\right)}\right)$ and $G_{200}=G\left(X^{\left(200\right)},A^{\left(200\right)}\right)$] are constructed for each subject based on two spatial scales.

#### 3.2.2. Multi-Scale Graph Representation Learning

Brain FCNs can be regarded as an irregular graph, internal structure and hence, we resort to spectral GCN (Zhang et al., 2018b) to analyze brain FCNs and learn new graph representations for ASD diagnosis in this work. Spectral GCN uses Fourier transform and inverse Fourier transform to realize the aggregation of information between nodes in the spectral space (Bruna et al., 2013; Kawahara et al., 2017).

The Fourier transform on the graph depends on the eigenvector of the Laplacian matrix. The Laplacian matrix (i.e., L) can be defined as (Bruna et al., 2013):


(2) $$\begin{array}{l} L=D-A \end{array}$$

where *D* is a diagonal matrix and its diagonal element *D*_*ii*_ represents the degree of the *i*-th node and *D*_*ii*_ = Σ_*j*_*A*_*ij*_.

A more common form of Laplacian matrix is the symmetric normalized Laplacian matrix:


(3) $$\begin{array}{l} L=D^{-\frac{1}{2}}LD^{-\frac{1}{2}}=I_{n}-D^{-\frac{1}{2}}AD^{-\frac{1}{2}} \end{array}$$

where *I*_*n*_ is an identity matrix.

The *L* can be eigen-decomposed to *UΛU*^*T*^. $U={\left\{u_{i}\right\}}_{i=1}^{n}$ represents orthogonal eigenvectors, $\Lambda =diag\left({\left\{\lambda_{i}\right\}}_{i=1}^{n}\right)$ is a diagonal matrix, and λ_*i*_ represents the eigenvalues of *u*_*i*_. Where, *U* can transform variables into spectral space for convolution operation on the graph.

The Fourier transform of signal convolution is equivalent to the product of signal Fourier transform (Shuman et al., 2013). Let *x*, *y* represent the signal (variable) of the node domain. The graph convolution can be defined as:


(4) $$\begin{array}{l} x*y=U\left(U^{T}y\right)\left(U^{T}x\right) \end{array}$$

where * represents the convolution operation.

Based on the above graph convolution definition (Equation 4), ChebyNet (Defferrard et al., 2016) is proposed for reducing computational complexity. Then, Kipf et al. further deduced the 1-order approximation of the *l*+1 layer network of ChebyNet for artificial convenience (Kipf and Welling, 2016):


(5) $$\begin{array}{l} H^{\left(l+1\right)}=\sigma \left({\tilde{D}}^{-\frac{1}{2}}\tilde{A}{\tilde{D}}^{-\frac{1}{2}}H^{\left(l\right)}W^{\left(l\right)}\right) \end{array}$$

where *H* represents the features of nodes on the graph, *W* is the network parameter to be learned, $\tilde{A}=A+I_{n}$, ${\tilde{D}}_{ii}=\Sigma_{j}{\tilde{A}}_{ij}$, and σ(·) is a non-linear activation function.

In this paper, we construct a GCN model with two-layer convolution for each scale graph, and the activation function of each layer convolution is ReLU. Then, the overall forward propagation formula is:


(6) $$\begin{array}{l} f\left(X,A\right)=ReLU\left[\tilde{A}ReLU\left(\tilde{A}XW^{\left(0\right)}\right)W^{\left(1\right)}\right] \end{array}$$

where, *f*(*X, A*)∈*R*^*n*×*d*^, *X*∈{*X*^(116)^, *X*^(200)^}, *A*∈{*A*^(116)^, *A*^(200)^}, *d* represents the output feature dimension. *W*^(0)^ is the weight parameter matrix of the 1-th convolution layer, and *W*^(1)^ is the weight parameter matrix of the 2-th convolution layer.

**Figure 2:**
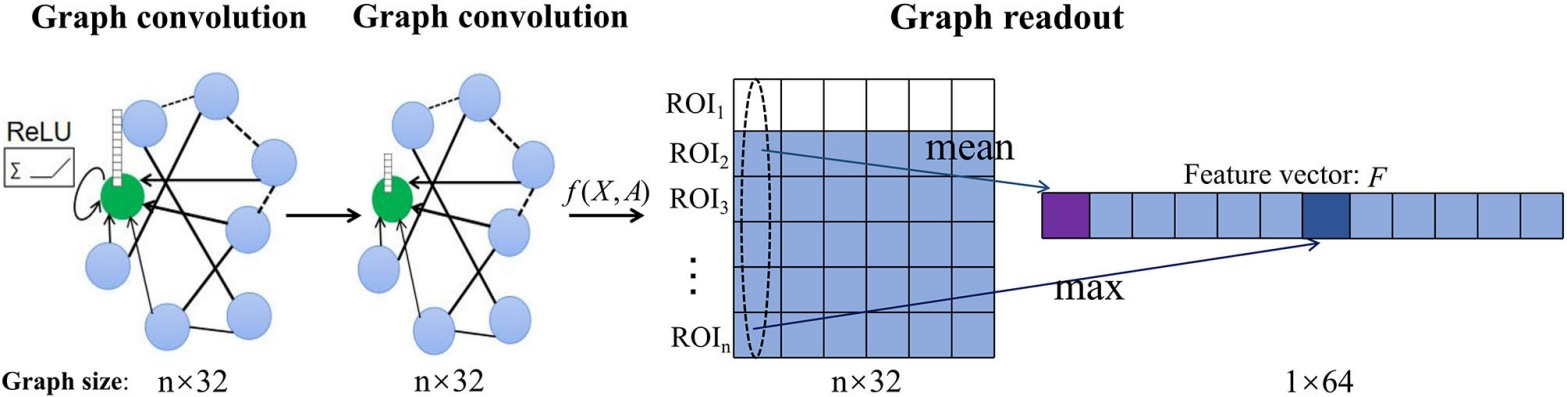
Illustration of the readout operation. Each graph convolution layer updates the features of the central node (green) by aggregating the information of connected nodes and non-linear activation of ReLU to obtain the feature matrix [*f*(*X, A*)] of each subject. Then, the graph-level vector representation (*F*) of each subject is summarized by maximum pool and average pool readout operations.

We use graph structure and node content information of different scales from the same subject as inputs to independently train GCN models of different scales so as to predict the category labels of the whole graph. The convolution layer is responsible for strict higher-order graph representation. After the convolution layer, the graph classification task usually needs to use the readout layer to read the graph level representation of the whole graph. Inspired by Lee et al., we use both maximum pool and average pool operations to aggregate node features and readout fixed-size graph representation vectors of two scales from the same subject (Lee et al., 2019). The readout layer is defined as:


(7) $$\begin{array}{l} F=\frac{1}{n}\overset{n}{\underset{i=1}{\sum }}f_{i}\left(X,A\right)||\underset{i=1}{max^{n}}f_{i}\left(X,A\right) \end{array}$$

where *f*_*i*_(*X, A*) is the feature vector of the *i*-th ROI obtained by the convolution operation and || denotes concatenation operation as illustrated in Figure 2.

As illustrated in Figure 1, finally, we extract the 64-dimensional graph representation vectors of different scale graphs: $F_{1}={\left[{F_{11}}^{\left(64\right)},{F_{21}}^{\left(64\right)},\cdots \;,{F_{N1}}^{\left(64\right)}\right]}^{\text{T}}$ and $F_{2}={\left[{F_{12}}^{\left(64\right)},{F_{22}}^{\left(64\right)},\cdots \;,{F_{N2}}^{\left(64\right)}\right]}^{\text{T}}$, where, *F*_*m*1_, *F*_*m*2_ represent the graph representation vector of different scales of the *m*-th subject and *m* = 1, 2, 3, ⋯ , *N*.

#### 3.2.3. Multi-Scale Feature Fusion and Classification

Here, we treat equally (through concatenation) the graph-level representation vector obtained through the above readout operation for subsequent multi-scale feature fusion. Then, the 128-dimensional new graph representation vector for each subject ($F_{1}\;||F_{2}={\left[{F_{1}}^{\left(128\right)},{F_{2}}^{\left(128\right)},\cdots \;,{F_{N}}^{\left(128\right)}\right]}^{\text{T}}$) is connected to three fully-connected layers and the feature information on the graph is further learned. The final output is sent to a Softmax layer for classification.

In the experiment, we will further study the influence of the combination of these two scale features in different proportions. Therefore, our model can automatically learn the topology information of the brain network through the convolution operation and make full use of the complementary information from the multi-scale FCNs of the same subject for classification.

### 3.3. Implementation Details

The proposed MGRL is implemented on Pytorch, with a GPU (NVIDIA GeForce RTX with 8 GB memory). The MGRL framework includes two GCN modules layers, three fully-connected layers, and a Softmax layer for prediction. Each GCN module is composed of two convolutional layers and a readout layer. Moreover, these two GCNs are independent of each other. Both the convolutional layers and the fully-connected layers are activated non-linearly by ReLU. The number of neurons in the two graph convolution layers is set as 32 and 32, respectively. The number of neurons in the three fully-connected layers are set as 128, 32, 16, respectively. The dropout for the fully-connected layers is 0.5. The Adaptive Moment Estimation (Adam) (Kingma and Ba, 2014) optimizer is used to optimize the model. The learning rate is 0.01, the regularization parameter is 0.00001, and the training epoch is 50.

## 4. Experiments

### 4.1. Experimental Settings

Considering the small amount of subjects, we randomly select 80% of all the samples as training data, 10% of all samples as validation data, and the remaining 10% as test data. We repeat the random partition process 100 times and record the mean and SD results of each method. For a fair comparison, we use the same data partitioning and model training strategies to evaluate our MGRL and the competing methods.

In order to evaluate the effectiveness of different methods, five metrics including accuracy, recall, precision, F1-score, and area under ROC curve (AUC) are used to evaluate the performance of the model. TP, TN, FP, and FN are denoted as True Positive, True Negative, False Positive, and False Negative, respectively. The first four metrics are defined as follows: Accuracy = $\frac{TP+TN}{TP+FN+FP+TN}$, Precision = $\frac{TP}{TP+FP}$, Recall = $\frac{TP}{TP+FN}$, and F1-Score = $\frac{2\times Precision\times Recall}{Precision+Recall}$. For these metrics, a higher value denotes that the corresponding model can achieve better classification performance. Besides, the Receiver Operating Characteristics (ROC) curve is composed of true positive rate (TPR, y-axis) and false positive rate (FPR, x-axis). The area under the ROC curve (AUC) is equal to the probability that the classifier will rank randomly selected positive cases higher than randomly selected negative cases, and the AUC value close to 1 is better.

### 4.2. Methods for Comparison

We compare the proposed MGRL approaches with three conventional networks representation methods and two GCNs based methods: (1) multi-scale feature fusion based on degree centrality (**DCF**), (2) multi-scale feature fusion based on local clustering coefficients (**LCCF**), (3) multi-scale feature fusion based on closeness centrality (**CCF**), (4) GCN with AAL atlas (**GCNA**), and (5) GCN with CC200 atlas (**GCNC**).

1) **Degree centrality**: This method uses DC to measure the node centrality that represents the FCN of each brain. That is, the greater the degree of the node, the higher the DC of the node, indicating that the node is more important in describing the network. Similar to MGRL, in this method, we first construct two fixed FC matrices (based on AAL and CC200 atlases) for each subject by calculating the PC coefficient between any pair of ROI time series (size: 116 × 116 and 200 × 200). We extract the DC value of each node for each specific FC matrix and then represent each FC network as a 116-dimensional (or 200-dimensional) feature vector based on a specific atlas. The two feature vectors are concatenated into a 316-dimensional feature vector to represent each subject, followed by 3 fully-connected layers for feature abstraction and a softmax layer for classification.
2) **Local clustering coefficients**: This method uses the local clustering coefficient (LCC) of the nodes to measure the degree of aggregation of each node on the FCNs with other nodes. That is, the larger the local clustering of a node, the stronger the correlation between the node and other nodes in the network. First, we use the PC coefficient of the BOLD signals in the same ROI to measure the pairwise correlation of the average time series of two ROIs and construct two FC matrices (based on AAL and CC200 atlases) for each subject (size: 116 × 116 and 200 × 200). We extract the LCC value of each node in each specific FC matrix and then represent each FCN as a 116-dimensional (or 200-dimensional) feature vector based on a specific atlas. Similar to the DCF method, a 316-dimensional feature vector is generated to represent each subject, followed by three fully-connected layers for feature abstraction and a softmax layer for classification.
3) **Closeness centrality**: This method uses closeness centrality (CC), which reflects the distance between an absolute node and other nodes in FC. First, we also use the PC coefficient to construct two FC matrices (size: 116 × 116 and 200 × 200) for each subject. Then, we calculate the shortest path distance from one node to all other nodes based on the calculations on these two FC matrices, respectively, and calculate the closeness centrality of each ROI. For a node/ROI, the closer it is to other nodes/ROIs, the greater its CC, and the greater the influence of this node/ROI on other nodes/ROIs in the network. Finally, the 116-dimensional and 200-dimensional feature vectors are obtained for each subject to represent each FCN, which are further concatenated into a 316-dimensional feature vector to represent each FCN/subject. The feature vector is further input to three fully-connected layers for feature extraction and a softmax layer for ASD diagnosis.
4) **GCN with AAL atlas**: The method uses the AAL atlas for ROI partitioning. Using AAL, the brain is divided into 116 interpretable ROIs and the average time series of the BOLD signals of the ROIs are extracted. We use the PC to calculate the pairwise correlation between brain signals and construct a FC matrix (size: 116 × 116) for each subject. In order to be comparable with the MGRL method, we use similar graph construction, graph convolution, and graph readout operations. The absolute value of the FC matrix is used as the adjacency matrix of the graph. The constructed graph is used as the input of the GCNA model to perform classification. The above models all include two graph convolutional layers, a readout layer and three fully-connected layers.
5) **GCN with CC200 atlas**: The method uses the CC200 atlas for ROI partitioning. Using the CC200 atlas, the brain is divided into 200 ROIs and the average time series of the BOLD signals of the ROIs are extracted. We also use PC to calculate construct an FC (size: 200 × 200) for each subject. Similar to GCNA, the GCNC method inputs each FC network to two graph convolutional layers, a readout layer, and three fully-connected layers for feature extraction and fusion, followed by a softmax layer for classification.

For three handcrafted FCN feature based methods (i.e., DCF, LCCF and CCF), the ReLU activation and 0.2 dropout are used after each fully-connected layer. The numbers of neurons in the three fully-connected layers are 316, 32, and 16, respectively. For two GCN-based methods (i.e., GCNA and GCNC), the number of neurons in the convolutional layers are set to 32 and 32, the number of neurons in three fully-connected layers are 64, 16, 8, respectively. Also, the classification is performed by the final layer (with two neurons) via Softmax. Note that the proposed MGRL and three handcrafted feature based methods (i.e., DCF, LCCF, and CCF) share the same multi-scale atlases (i.e., AAL and CC200) for ROIs partition, as well as the same multi-scale feature fusion strategy (i.e., multi-scale concatenation followed by three fully-connected layers). Besides, two GCN-based methods (i.e., GCNA and GCNC) use a single atlas and share the same network architecture as MGRL. For a fair comparison, five competing methods use the Adam optimizer and cross-entropy loss for network training. The learning rate is set to 0.01, the regularization parameter is 0.00001, while the training epoch is 50.

### 4.3. Classification Results

**Figure 3:**
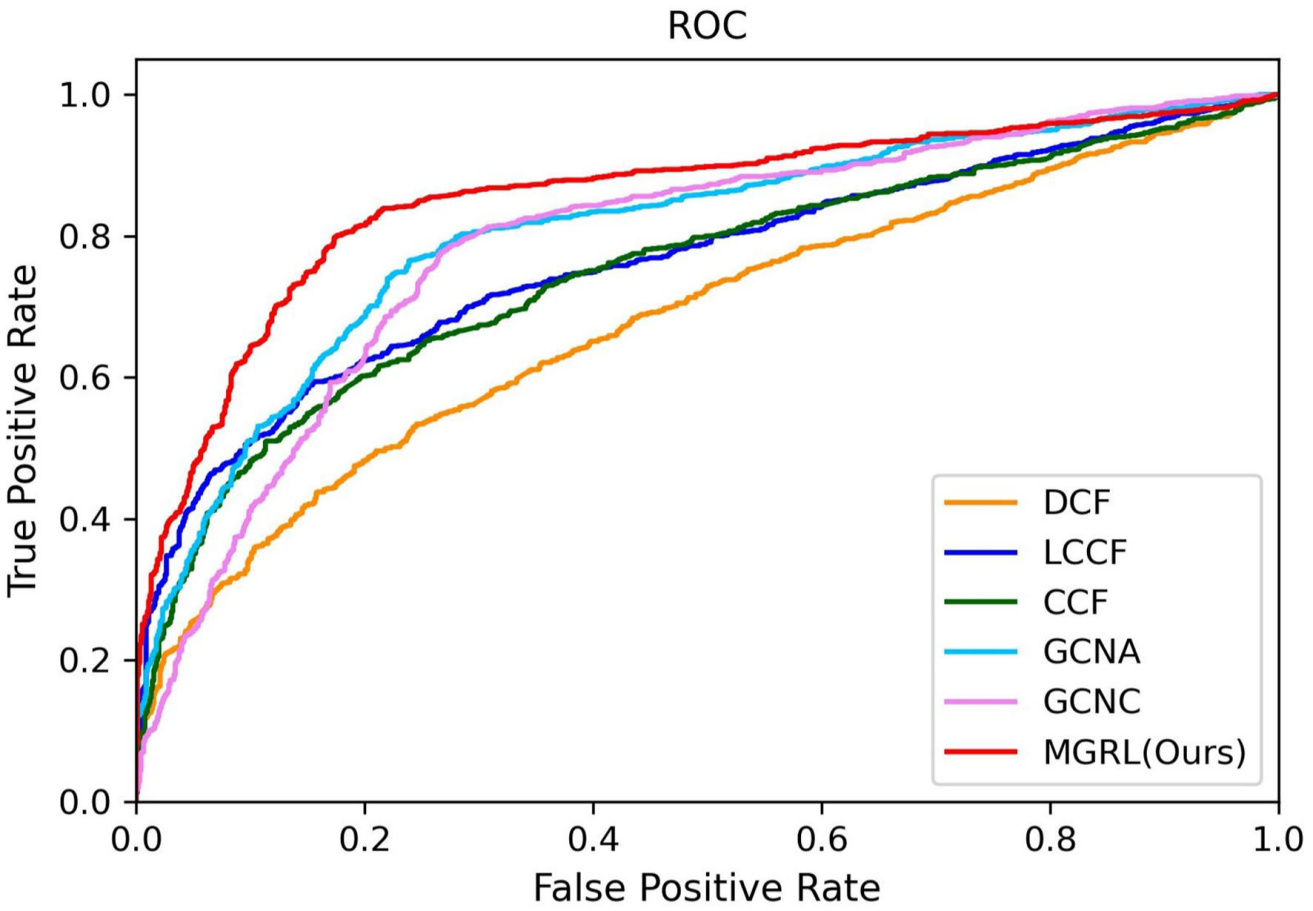
ROC curves achieved by six different methods in ASD vs. normal controls (NC) classification.

The quantitative results of our MGRL and five competing methods in ASD vs. NC classification are reported in Table 2, while the ROC curves of these methods are shown in Figure 3. From Table 2 and Figure 3, one can have the following interesting observations. *First*, the proposed MGRL achieves the overall best results in ASD vs. NC classification in terms of five metrics and ROC curve, compared with five competing methods. *Second*, compared with two single-scale GCN methods (i.e., GCNA and GCNC), the MGRL improved the classification performance by at least 3% in terms of accuracy, F1-score, recall, precision, and AUC. These results suggest that using multi-scale brain atlases helps boost the classification performance, when compared with that using a single atlas. The underlying reason is that FCNs features learned at different spatial scales may contain complementary information that can be collaboratively used to improve the classification results. *Besides*, among four multi-scale methods, the proposed MGRL method generally outperforms the other three methods (i.e., DCF, LCCF, and CCF) in terms of five evaluation metrics. This further demonstrates the advantage of deep learning models in ASD diagnosis by jointly performing FCNs feature learning and classification.

**Table 2:** Performance (mean ± SD) of different models in autism spectrum disorder (ASD) vs. normal control (NC) classification based on resting-state functional MRI (rs-fMRI) data in NYU site of the ABIDE dataset.

**Model**	**Accuracy**	**Recall**	**Precision**	**F1-score**	**AUC**
DCF	0.624 ± 0.060	0.703 ± 0.197	0.676 ± 0.142	0.652 ± 0.076	0.711 ± 0.071
LCCF	0.705 ± 0.085	0.676 ± 0.196	0.819 ± 0.171	0.698 ± 0.102	0.812 ± 0.033
CCF	0.672 ± 0.111	0.781 ± 0.153	0.724 ± 0.173	0.719 ± 0.060	0.792 ± 0.039
GCNA	0.758 ± 0.075	0.761 ± 0.146	0.789 ± 0.089	0.763 ± 0.087	0.854 ± 0.058
GCNC	0.753 ± 0.080	0.773 ± 0.171	0.759 ± 0.100	0.757 ± 0.120	0.808 ± 0.074
MGRL (Ours)	**0.795 ± 0.068**	**0.809 ± 0.146**	**0.823 ± 0.103**	**0.802 ± 0.075**	**0.886 ± 0.062**

*The bold values mean to highlight the experiment results*.

To further test the robustness of the MGRL model, we also perform ASD vs. NC classification on the University of Michigan (UM) site from ABIDE. The phenotype information of UM site is in Table 1, and the experimental results are in Table 3. The results in Table 3 suggest that our MGRL outperforms five competing methods (GCNA, GCNC, DCF, LCCF, and CCF) on the UM site, in terms of accuracy, F1-score, and recall. The underlying reason is that FCN features learned at different spatial scales may contain complementary information, which can be used together to improve the classification results.

**Table 3:** Performance (mean ± SD) of different models in ASD vs. NC classification based on rs-fMRI data in UM site of the ABIDE dataset.

**Model**	**Accuracy**	**Recall**	**Precision**	**F1-score**	**AUC**
DCF	0.645 ± 0.089	0.733 ± 0.187	0.691 ± 0.046	0.702 ± 0.103	0.633 ± 0.039
LCCF	0.620 ± 0.077	0.720 ± 0.200	0.670 ± 0.059	0.681 ± 0.116	0.573 ± 0.045
CCF	0.634 ± 0.105	0.761 ± 0.190	0.662 ± 0.076	0.702 ± 0.131	0.542 ± 0.049
GCNA	0.739 ± 0.098	0.710 ± 0.159	**0.927 ± 0.067**	0.790 ± 0.102	**0.875 ± 0.065**
GCNC	0.725 ± 0.082	0.651 ± 0.186	0.751 ± 0.108	0.677 ± 0.121	0.824 ± 0.057
MGRL (Ours)	**0.762 ± 0.078**	**0.843 ± 0.070**	0.794 ± 0.100	**0.812 ± 0.054**	0.867 ± 0.049

*The bold values mean to highlight the experiment results*.

### 4.4. Visualization of Network Features

**Figure 4:**
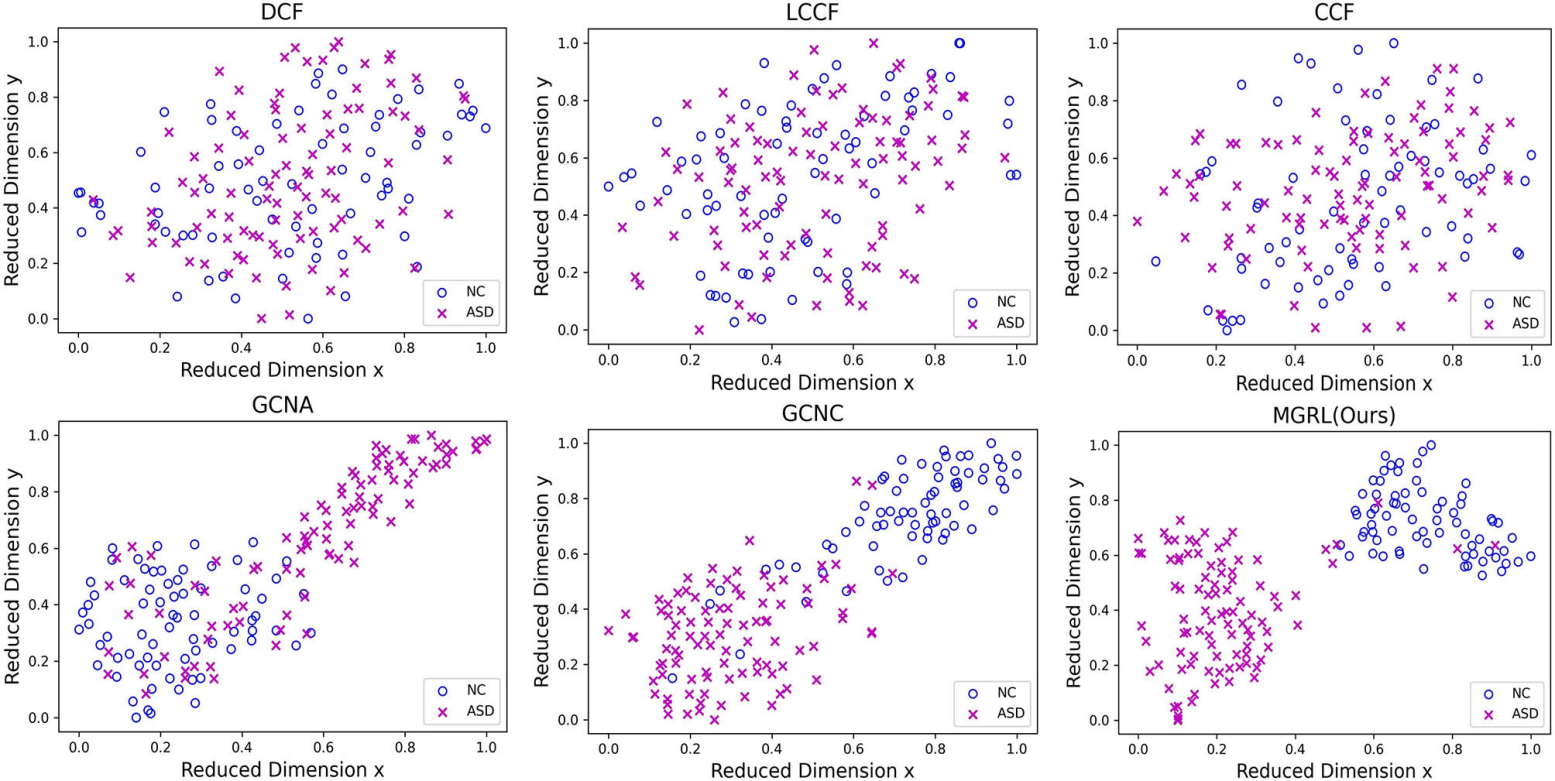
Manifold visualization of ASD and NC training subjects in the New York University (NYU) site, where t-SNE (Van der Maaten and Hinton, 2008) is used to project the multi-scale graph representations of FCNs learned by six different models. Note that the original features of three GCN-based methods (i.e., GCN with AAL atlas, GCNA; GCN with CC200 atlas, GCNC; and multi-scale graph representation learning, MGRL) are generated by graph representation learning, while three conventional methods (i.e., DCF, LCCF, and CCF) use the concatenation of two-atlas features (i.e., degree centrality, local clustering coefficient, or closeness centrality features), respectively.

To investigate the distributions of FCNs features learned by different methods, we use the t-SNE (Van der Maaten and Hinton, 2008) algorithm to reduce the dimensionality of FCN features of six methods (i.e., DCF, LCCF, CCF, GCNA, GCNC, and MGRL) to two dimensions, with results shown in Figure 4. Note that the FCN features of three GCN-based methods (i.e., GCNA, GCNC, and MGRL) are generated by graph representation learning, while three conventional methods (i.e., DCF, LCCF, and CCF) use the concatenation of two-atlas features (i.e., degree centrality, local clustering coefficient, or closeness centrality features). As can be seen from Figure 4, with three GCN-based methods (i.e., GCNA, GCNC, and our MGRL), samples of different categories tend to be as far as possible, while those of the same category tend to be as close as possible. But this trend is not obvious for three conventional methods (i.e., DCF, LCCF, and CCF). This implies that graph convolution operation used in GCN-based methods help extract more discriminative features for ASD detection, compared with three conventional methods.

## 5. Discussion

### 5.1. Influence of FCN Construction

**Figure 5:**
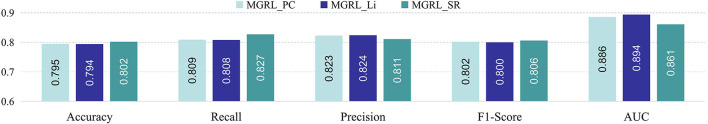
Results of three methods (with different network construction strategies) based on NYU site for identifying ASD from NC.

In the experiments, we use Pearson's correlation (PC) for FCNs construction. To investigate the influence of different FCN construction strategies, we compare our MGRL with two additional methods: (1) **MGRL_Li** uses the method proposed by Li et al. for FCNs construction Li et al. (2017), (2) **MGRL_SR** employs the sparse representation (SR) method Qiao et al. (2016) for FCNs construction. For clarity, we denote our MGRL with PC as **MGRL_PC** here. For fair comparison, these three methods share the same network architecture and parameter settings, and they differ only in FC construction strategies. In Figure 5, we report the results of three methods in ASD vs. NC classification. This figure suggests that three methods achieve comparable results, while MGRL_SR is superior to MGRL_PC and MGRL_Li in terms of accuracy, recall, and F1-score. The underlying reason is that the SR algorithm can generate much sparser and less noisy brain FCNs, compared with the other two methods.

**Figure 6:**
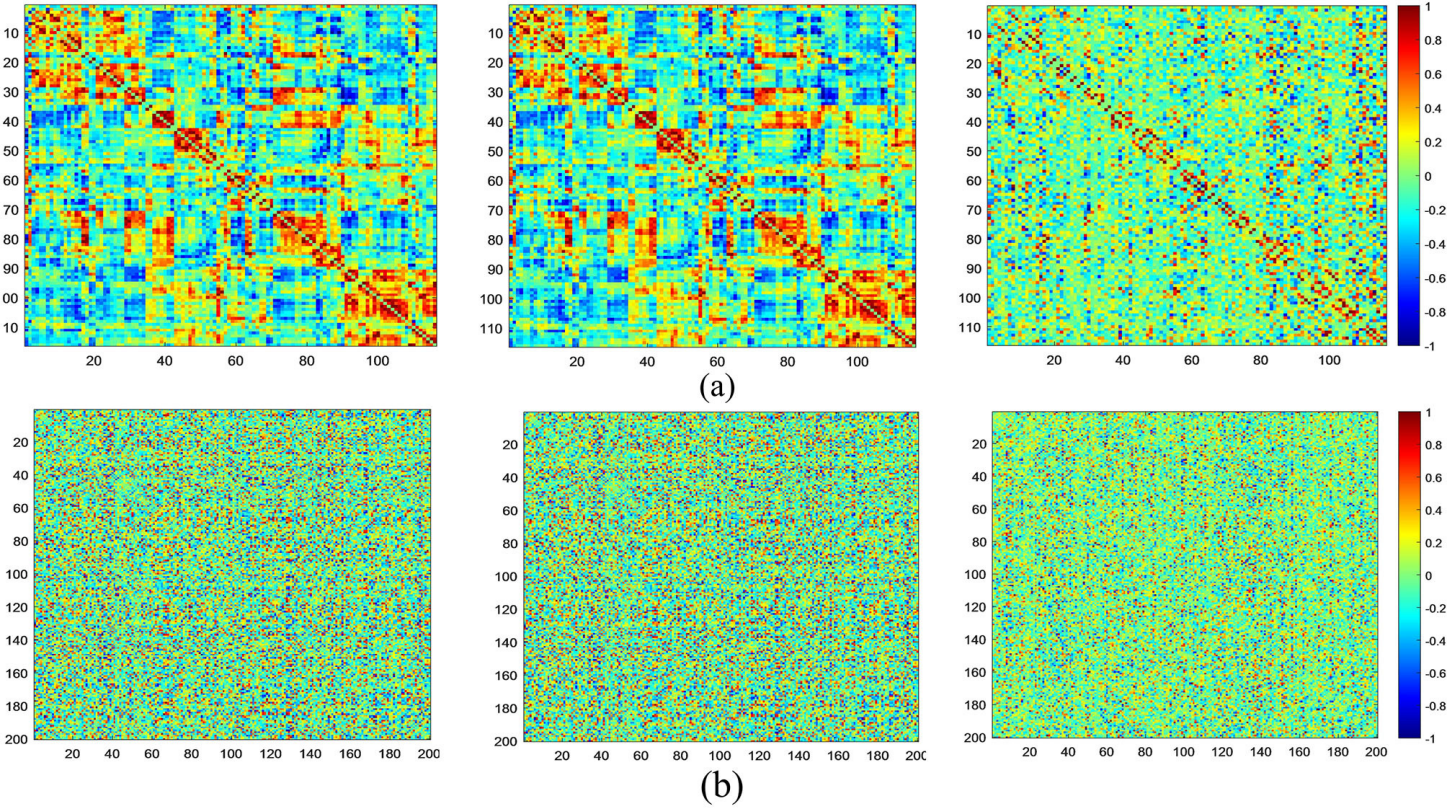
Visualization of FCNs for the same subject (NYU_50952) constructed by MGRL_PC (1st row), MGRL_Li (2nd row), and MGRL_SR (3rd row) based on the AAL atlas **(a)** and the CC200 atlas **(b)**.

At the same time, in Figure 6, we visualize FCNs constructed via three models based on both the AAL (Figure 6a) and CC200 (Figure 6b) atlases. From this figure, we can see that compared with the other two methods, MGRL_SR can generate brain FCNs where functional connections among different ROIs tend to be sparser.

**Figure 7:**
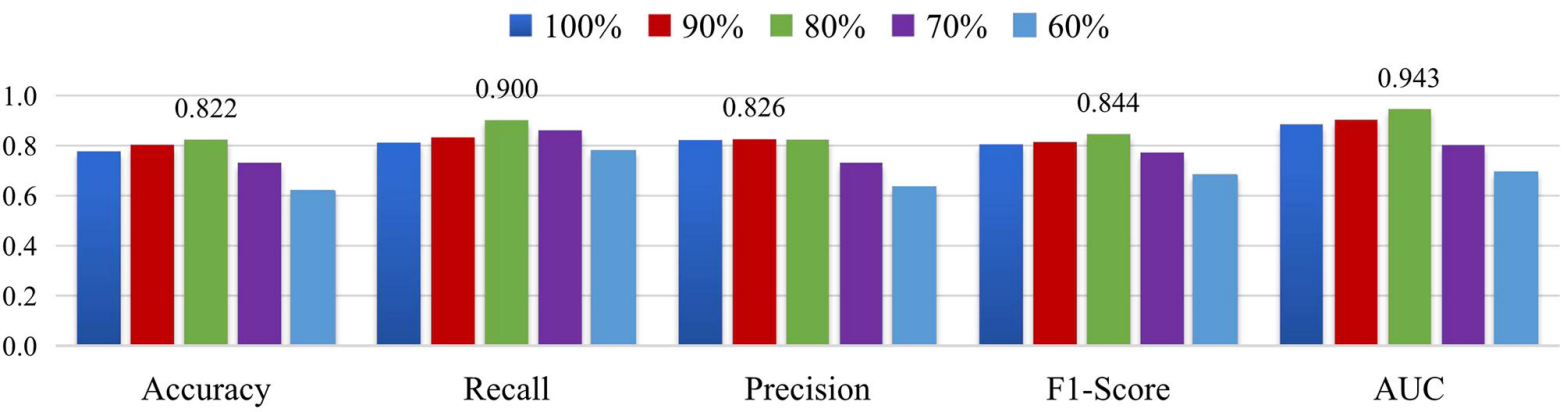
Results of MGRL_PC in ASD vs. NC classification when retaining 100%, 90%, 80%, 70% and 60% functional connections of original brain FC networks on the NYU site.

In addition, we select the threshold corresponding to the different sparsity in the set [60, 70, 80, 90, 100%] for multi-scale FCNs of MGRL_PC, where the percentage indicates the proportion of the edges that are retained. Then, we perform ASD vs. NC classification on the NYU site. The experimental results are reported in Figure 7. As shown in Figure 7, the MGRL_PC model with 80% brain FC achieves the best performance in terms of accuracy, recall, F1-score, and AUC. This implies that brain FCN may contain some noisy/redundant connections that may negatively affect the performance of the model.

### 5.2. Influence of Atlas Fusion

**Figure 8:**
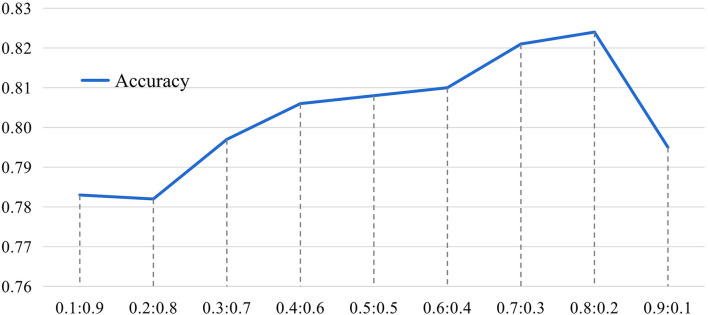
The results of the influence of different proportions of AAL and CC200 brain atlas on the classification accuracy of our MGRL model based on the NYU site during feature information fusion. The horizontal axis represents the proportion of AAL atlas and CC200 atlas features in fusion. The ordinate represents model classification accuracy.

In the proposed MGRL, two brain atlases (i.e., AAL and CC200) are used for ROI partition, and the generated FCN features are equally treated and fused for classification. We now investigate the influence of these two spatial scales when performing multi-scale feature fusion by varying the ratio of AAL to CC200 within the range of $\left\{\frac{0.1}{0.9},\frac{0.2}{0.8},\frac{0.3}{0.7},\cdots \;,\frac{0.9}{0.1}\right\}$, with experimental results reported in Figure 8.

It can be seen from Figure 8 that the fusion ratio has a significant impact on the classification accuracy of the proposed MGRL in ASD diagnosis. As the ratio increases, the accuracy value gradually rises, and the best accuracy is obtained when the fusion ratio of AAL to CC200 is $\frac{0.8}{0.2}$. This may be because the AAL atlas is a functional template divided according to the data of brain structure items, which is more consistent with our cognition and more beneficial to ASD diagnosis. In addition, these results also suggest that the fusion of multi-scale FCNs features does improve the learning performance compared to using only a single atlas for ROI partition.

**Figure 9:**
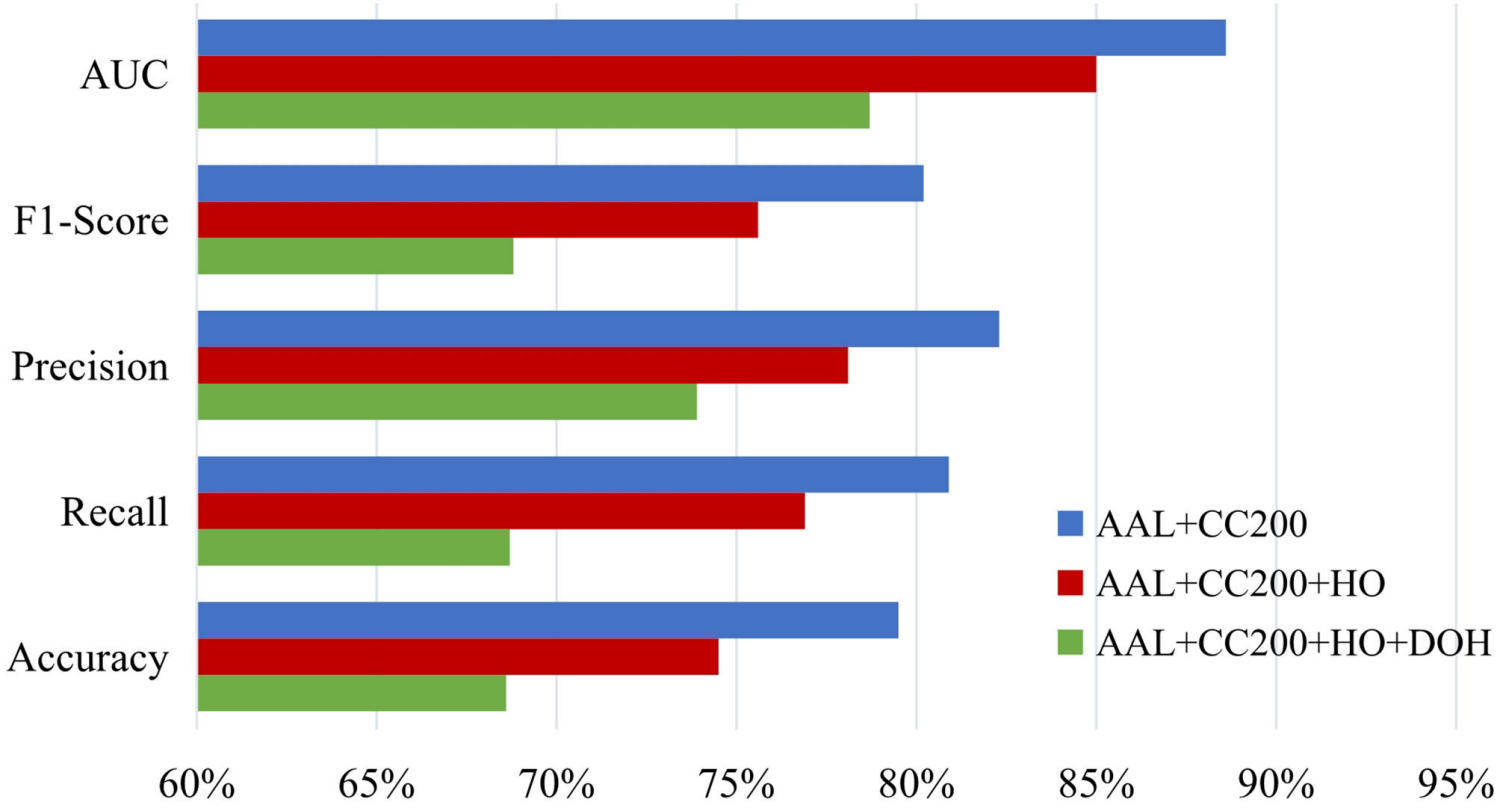
Results of the proposed MGRL method based on NYU site using multi-scale atlases for ROI partition in the task of ASD vs. NC classification. HO, Harvard Oxford atlas with 112 ROIs; DOH, Dosenbach atlas with 160 ROIs (Dosenbach et al., 2010).

Besides, we also study the influence of the number of atlases and report the ASD diagnosis results of our MGRL method with two (i.e., AAL and CC200), three (i.e., AAL, CC200, and HO), and four (i.e., AAL, CC200, HO and Dosenbach) atlases in Figure 9. This figure suggests that MGRL with two atlases achieves the best results and adding more atlases does not boost the performance. The possible reason is that high-dimensional node features obtained from multiple atlases may contain redundant or noisy information, thus reducing the classification performance.

### 5.3. Comparison With State-Of-The-Art

We further compared the results of the MGRL experiment with several state-of-the-art methods for fMRI-based ASD diagnosis based on the ABIDE database. Specifically, Sun et al. proposed an FCNs estimation model (without hyperparameters) to avoid the parameter selection problem and used the traditional support vector machine (SVM) (Cortes and Vapnik, 1995) for classification (Sun et al., 2021). Wang et al. proposed a multi-site domain adaptation framework with low-rank representation and used traditional SVM classifier to identify ASD (Wang et al., 2019b). The AAL atlas with 116 brain regions was used for the above-mentioned two methods. Parisot et al. and Cao et al. employed fMRI data and phenotypic information of ROIs based on the HO atlas and constructed a GCN model to identify ASD from NC (Parisot et al., 2018; Cao et al., 2021). Shrivastava et al. used the Craddock 400 (CC400) (Desikan et al., 2006) atlas for ROI partition and developed a convolutional neural network (CNN) for ASD diagnosis (Shrivastava et al., 2020). Niu et al. proposed to take advantage of complementary information provided by different brain atlases and designed a multi-channel Deep Attention Neural Network (DANN) model for automated ASD diagnosis (Niu et al., 2020). The classification results of our MGRL and these state-of-the-art methods are given in the Table 4. Note that the results in this table are not fully comparable, since different studies use different subsets of ABIDE. The rough comparison in Table 4 demonstrates the superiority of the MGRL method in mining and fusing multi-scale FCNs features for ASD diagnosis.

**Table 4:** Performance comparison between the proposed MGRL and several state-of-the-art methods for ASD vs. NC classification with fMRI data from ABIDE.

**Method**	**Algorithm**	**Accuracy**	**Recall**	**Precision**	**AUC**	**Sample #**	**Brain Atlas**
MGRL (Ours)	GCN	**0.795**	**0.809**	**0.823**	**0.886**	184	AAL, CC200

*The results of multi-scale graph representation learning (MGRL) are based on the NYU site. ^#^represents the number of samples. The bold values mean to highlight the experiment results*.

### 5.4. Limitations and Future Work

To further improve the performance of the proposed method, several technical issues need to be considered. *On the one hand*, to avoid significant inter-site heterogeneity, we mainly used rs-fMRI data from the NYU site. While ABIDE contains data from 21 sites, as a future work, we will evaluate the proposed method on all sites in ABIDE and design smart techniques to reduce inter-site data heterogeneity. *On the other hand*, the direct fusion of multi-scale FCN features may introduce redundant or even noise information, thus degrading the learning performance. Interestingly, incorporating the high-level attention mechanism into the current framework can avoid the negative impact of redundant/noisy information, which is also our next consideration.

## 6. Conclusion

In this paper, we develop a multi-scale graph representation learning (MGRL) framework for the automatic diagnosis of ASD based on rs-fMRI. In MGRL, for each subject, we first use multiple brain atlases for ROI partition to construct multi-scale FCNs. Then, we employ multi-scale GCNs for FCNs feature learning, followed by feature fusion and classification. We evaluate the MGRL on 184 subjects from ABIDE database with rs-fMRI scans, with results demonstrating its effectiveness in FCNs feature learning and ASD diagnosis.

## Data Availability Statement

The original contributions presented in the study are included in the article/supplementary material, further inquiries can be directed to the corresponding author/s.

## Ethics Statement

Ethical review and approval was not required for the study on human participants in accordance with the local legislation and institutional requirements. Written informed consent from the participants' legal guardian/next of kin was not required to participate in this study in accordance with the national legislation and the institutional requirements.

## Author Contributions

YC and ML designed the study. YC downloaded and analyzed the data, performed the experiments, and drafted the manuscript. YC, GW, LC, LQ, and ML revised the manuscript. All authors have read and approved the final manuscript.

## Funding

YC, GW, and LQ were partly supported by National Natural Science Foundation of China (Nos. 62176112, 61976110, and 11931008), Natural Science Foundation of Shandong Province (Nos. ZR2018MF020 and ZR2019YQ27), and Taishan Scholar Program of Shandong Province and the Open Project of Liaocheng University Animal Husbandry Discipline (No. 319312101-01).

## Conflict of Interest

The authors declare that the research was conducted in the absence of any commercial or financial relationships that could be construed as a potential conflict of interest.

## Publisher's Note

All claims expressed in this article are solely those of the authors and do not necessarily represent those of their affiliated organizations, or those of the publisher, the editors and the reviewers. Any product that may be evaluated in this article, or claim that may be made by its manufacturer, is not guaranteed or endorsed by the publisher.
